# The Assessment of Dry Eye Disease in Incense Users: A Pilot Cross-Sectional Study Integrating Clinical and Tear Biomarker Analysis

**DOI:** 10.3390/healthcare14101351

**Published:** 2026-05-14

**Authors:** Amani Y. Alhalwani, Ali S. Alsudais, Abdulaziz S. Alrashid, Salma Hamdan Almarwani, Qusay Aloweiny, Mohammed Basendwah, Alaa Hesham Mofti, Muhammad Anwar Khan

**Affiliations:** 1 College of Science and Health Professions, King Saud Bin Abdulaziz University for Health Sciences, Jeddah 21423, Saudi Arabia; 2 Department of Biomedical Research, King Abdullah International Medical Research Center, Jeddah 22384, Saudi Arabia or dr.amofti@hotmail.com (A.H.M.); khana@ksau-hs.edu.sa (M.A.K.); 3 College of Medicine, King Saud Bin Abdulaziz University for Health Sciences, Jeddah 21423, Saudi Arabia; a.alsudais00@gmail.com (A.S.A.); alrashidabdulazizs@gmail.com (A.S.A.); 421220124@ksau-hs.edu.sa (S.H.A.); aloweiny230@ksau-hs.edu.sa (Q.A.); 4 Department of Ophthalmology, Ministry of the National Guard-Health Affairs, Jeddah 21423, Saudi Arabia; dr.mohdbas@yahoo.com

**Keywords:** dry eye disease, tear break-up time, inflammation, schirmer II tests, the ocular comfort index, tear proteins

## Abstract

Background: Dry eye disease (DED) is a multifactorial disease. Numerous risk factors might cause DED, including indoor air pollution, such as incense. Incense (Bakhoor) is widely used in many cultures, including Saudi Arabia, although its smoke contains toxic chemicals that pose serious health hazards. This research investigates the link between the Schirmer II test and tear fluid proteins in DED patients. The study focuses on identifying the ocular examinations, hypothesizing that incense smoke, particularly from synthetic types, exacerbates DED. Methods: This pilot cross-sectional study was conducted at King Abdulaziz Medical City (KAMC) in Jeddah, Saudi Arabia. Participants were recruited from the Cornea and Ophthalmology Clinics. Eye assessments analyzed tear protein concentrations, including tear collection using Schirmer II test strips and tear break-up time (TBUT). The study included DED patients who used incense. Tear fluid from the Schirmer test of 20 randomly selected patients was used for protein analysis of total protein, lactoferrin, and Immunoglobulin E. Inclusion criteria were male and female subjects aged 18 years or older, diagnosed with DED, and using incense. The sample size was 55 participants, selected via convenience sampling. Subjective data were collected through questionnaires, as well as objective data from the tear test and the sample and analyzed with SPSS. Descriptive and inferential statistics were used, with statistical significance set at *p*-value < 0.05. Results: The Ocular Comfort Index (OCI) categories showed that 21.8% had no symptoms, 40.0% had low symptoms, 30.9% had moderate symptoms, and 7.3% reported high symptoms. TBUT values and Schirmer test scores decreased with increasing OCI severity, with no statistical significance. The mean (SD) of total protein in the right and left eyes for high OCI was 7.19 (1.39) and 7.42 (0.91), respectively, with no statistical significance. The immunoglobulin E levels in the right and left eyes for high OCI were 301.71 (55.97) and 301.71 (47.14), respectively, with no statistical significance. The mean (SD) of lactoferrin in the right and left eyes for high OCI was 163.77 (10.42) and 159.43 (1.68), respectively, with no statistical significance. Conclusions: The study findings demonstrate alignment in incense-using patients between subjective OCI symptom scores and objective clinical diagnostic measures. Specifically, higher OCI scores are associated with lower TBUT and Schirmer II test values, as well as changes in tear biomarkers such as IgE and lactoferrin. These findings emphasize the potential of using simple screening methods combined with bioanalytical markers for early detection of ocular surface disease. This highlights the potential health risks associated with incense exposure, particularly for individuals predisposed to DED. The urgency for further research to explore the long-term effects of incense on ocular health and to raise awareness about its potential impact on populations with high incense usage cannot be overstated.

## 1. Introduction

Dry eye disease (DED) is a multifactorial disease of the ocular surface characterized by a loss of tear film homeostasis, ocular discomfort, visual disturbance, and surface inflammation [1]. DED commonly presents symptoms such as itching, burning, stinging, dryness, a foreign-body sensation, photophobia, and transient blurred vision, all of which significantly impair quality of life [1]. To evaluate the severity of these symptoms, a comprehensive symptom assessment includes DED diagnosis, and clinicians often use validated questionnaires as a subjective clinical assessment, such as the Ocular Surface Disease Index (OSDI) [2] and the Ocular Comfort Index (OCI) [3]. The OCI measures the extent to which patients feel irritated on the ocular surface and provides insight into an individual’s symptoms over time [3]. Additionally, objective clinical assessments for DED diagnosis, including tear break-up time (TBUT) and the Schirmer II test, are routinely employed; TBUT evaluates tear film stability, with values under 10 s (Sec.) considered abnormal, while the Schirmer II test measures basal tear secretion, with readings below 10 milliliters (mm) in two minutes indicating dry eye [4].

Various risk factors have been linked to DED, such as age, female gender, contact lens use, screen exposure, systemic medications, autoimmune diseases, smoking, environmental pollutants, geographical location, and air pollution outdoors and indoors [5]. Indoor air pollution, most notably by incense smoke, particularly dried flower incense such as Bakhoor, a traditional aromatic resin burned in Middle Eastern and South Asian cultures, has recently emerged as a potential environmental risk factor. Incense combustion produces particulate matter and volatile organic compounds that may contribute to ocular surface irritation, inflammation, and tear film instability [5]. Particulate matter (PM) can lead to visual symptoms such as tears and burning, increased mucus, conjunctivitis, conjunctival edema, and itching [5,6,7]. Among the latter, incense burning has been gaining growing attention, as it has been shown to emit substantial levels of fine particulate matter in enclosed indoor environments, often exceeding established air quality thresholds [8], and has been identified through stable-isotope analysis as a primary indoor particulate matter source [9]. This is particularly concerning given that PM_2.5_ exposure is known to trigger oxidative stress and destabilize the tear film, thereby exacerbating dry eye symptoms [8]. Another chemical found in incense is nitrogen dioxide (NO2). Exposure to high concentrations of nitrogen dioxide can irritate the eyes, and prolonged exposure can cause cloudiness in the eyes, which, if persistent, can lead to blindness. Because of these harmful effects, certain inhabitants of the world, such as those in Asia and the Middle East, where there is high consumption of incense, should be aware of the extensive use of incense and its complications for the eyes [10,11].

Several studies have suggested that air pollutants, including incense smoke, can have adverse effects on the ocular surface. A study by Mandell et al. (2020) highlighted the utility of tear film tests in evaluating ocular surface changes caused by environmental irritants [12]. Afonso et al. (1999) correlated Schirmer test scores with symptom severity in polluted environments [13]. A recent high-impact publication in the American Journal of Ophthalmology demonstrated significant associations between indoor pollutants, including incense, and increased prevalence of ocular surface disorders, underscoring the importance of environmental control in DED management [14].

The chemical components released by burning incense affect internal systems such as the respiratory system, but they can also have an indirect effect on the human eyes, leading to the development of dry eye disease in incense users [8]. Chemical elements in incense, such as nitric dioxide and particulate matter, can cause eye surface inflammation and have a negative impact on eye tissues. These chemical pollutants have attracted more attention, and a considerable number of studies have correlated the impact of these chemicals on harming human eye health.

The purpose of the present research was to investigate factors that affect the toxicity of incense to evaluate its potential health risks.

The relationship between incense use and ocular health is particularly important in areas where Bakhoor is culturally prevalent and frequently burned indoors [11,15]. Extended or repeated exposure can worsen or cause dry eye symptoms, especially in poorly ventilated spaces. Understanding this link is essential for creating public health guidelines, particularly for populations with a high incidence of use [16].

Despite these findings, a notable gap remains in the literature regarding the direct assessment of incense (Bakhoor) use and its specific impact on ocular health, particularly using comprehensive tools such as the OCI, TBUT, and Schirmer II test in community-based populations. Most existing studies either address general air pollution or focus on respiratory health, leaving ocular health underexplored.

Our previous study has recommended enhancing understanding of ocular surface disease and reducing potential health risks associated with incense exposure through ocular examinations and tear sample analyses, and has advised moderate incense use to reduce ocular health risks [17]. This study aimed to fill this gap by conducting a pilot cross-sectional study of incense users’ ocular surface health. It combined subjective assessments of ocular symptoms (OCI) with objective clinical diagnostics, including TBUT and Schirmer II tests, as well as tear analysis for specific biomarkers. The clinical diagnostics data served as a screening tool to quantify symptom severity, allowing comparison with clinical and biochemical data. The aim was to investigate evidence-based information to inform clinical screening protocols and public health strategies, particularly in regions where incense use is common.

## 2. Methodology

### 2.1. Study Population

A pilot cross-sectional study among incense users was conducted at the ophthalmology outpatients clinic of King Abdulaziz Medical City (Jeddah, Saudi Arabia) from August 2023 to October 2024. Through a convenience sample, 55 patients were selected based on their willingness to be in the study. The prospective study applies to OCI interview-administered questionnaires, TBUT, and the Schirmer II test. The sample size was determined by the number of available participants who contributed to this study and were willing to undergo testing. Inclusion criteria were male and female subjects aged 18 years or older, diagnosed with DED based on clinical assessment, and incense use status. Additionally, participants who underwent vision-correction surgery with a high cause of dry eye symptoms, such as LASIK, or had glaucoma or other chronic diseases besides DED, such as rheumatoid arthritis, lupus, scleroderma, Sjogren’s syndrome, cancer, thyroid disorders, vitamin A deficiency, cardiovascular disease, and hypertension, were excluded from the study. However, participants with other chronic diseases, such as asthma and anemia, were reported during the screening.

### 2.2. Ocular Comfort Index (OCI)

Patients’ ocular health was assessed using the Ocular Comfort Index (OCI) and tested for dry eye disease (right eye and left eye) using the Schirmer II test and TBUT. Given that this is a pilot study, testing was conducted on both eyes, including screening and tear samples, to identify potential asymmetries in ocular surface health and tear film composition. It was essential to verify that the biological markers were consistent across both eyes in clinical assessment to ensure the tests’ diagnostic reliability.

OCI is employed in this study due to its advantages in ocular health assessment [3]. The focus of this study requires questions regarding the OCI and evaluation of incense users. The patients were divided into four categories based on their OCI scores as follows: do not feel (0), low (1–8), moderate (9–16), and high (17–24), which was defined as having symptoms and at least one positive objective sign.

### 2.3. Ocular Examination Using Schirmer II Test and Tear Break-Up Time (TBUT)

The diagnostic tests, including the ocular examination, TBUT, and Schirmer II test, were performed at the ophthalmology outpatient clinic.

First, the Schirmer II test was applied to measure tear production for dry eye disease assessment. This test was utilized under local anesthesia, and tear fluid was examined from the fornix using Schirmer paper strips. Schirmer paper strips were placed in the patients’ fornix for 2 min, and the collected tear fluid measurements were documented for further analysis. Schirmer II test scores of ˂10 mm points were considered dry eye disease [13]. Additionally, the tear fluid was collected from Schirmer paper strips, which were diffused in MilliQ water to extract the protein, mixed quickly to facilitate dispersion of the tears into the water, and ultimately frozen at −80 °C for preservation until analysis is conducted. The final tear sample is diluted from the original collected tear sample.

Second, the diagnosis was based on utilizing TBUT in participants as an ocular examination at the ophthalmology clinic, measuring eye tear stability. TBUT scores of ˂10 s were considered dry eye disease [18].

### 2.4. Tear Sample Analysis

A random sample of 20 patients (40 eyes) was selected from the total Schirmer tear samples to measure tear chemical levels of various biomarkers. All tear samples were collected using Schirmer paper strips and tested according to the manufacturer’s instructions while considering the dilution factors, employing the lactoferrin (LF) enzyme-linked immunosorbent assay for quantitative detection of LF (as it serves as a key diagnostic biomarker for its anti-inflammatory and antimicrobial effects, and is a primary indicator of ocular surface health), Catalog Number EK710383; the results revealed a dilution factor of 5. The Immunoglobulin E (IgE) enzyme-linked immunosorbent assay was used for quantitative detection of IgE (as a primary biomarker for allergy and an indicator of allergic response, used to investigate the impact of environmental air exposure on the ocular surface), Catalog Number BMS2097; the results revealed a dilution factor of 10. The bicinchoninic acid (BCA) assay was used for quantitative detection of total proteins (as a baseline indicator to evaluate the overall protein concentration), Catalog Number 23225; the results revealed a dilution factor of 21.

### 2.5. Data Collection and Analysis

The patient’s demographic data, including age (years), gender (male and female), medical history, and DED diagnosis, were obtained from the hospital information system BestCare according to the World Health Organization’s International Classification of Diseases, 10th edition codes listed in the patient’s medical records.

### 2.6. Statistical Analysis

According to the manufacturer’s manual, all statistical analyses were performed using the SPSS complex samples procedure (SPSS Statistics for Windows, Version 20.0. Armonk, NY, United States.: IBM Corp (2011). For descriptive statistics, frequency (*n*) and percentage (%) were computed for categorical variables such as gender, severity level, smoking, and incense user evaluation parameters. Mean and standard deviation (SD) were estimated for quantitative variables, including age, TBUT level, and Schirmer II test (median and interquartile range for skewed data). Frequencies/percentages were shown in bar charts/tables, and tables/box plots will be displayed for mean and standard deviation. For inferential statistics, the Chi-square test was used to compare two categorical variables: severity level and incense user evaluation parameters. In contrast, an ANOVA was used to compare qualitative and quantitative variables. All statistical tests are considered significant with a *p*-value ≤ 0.05.

## 3. Results

A total of 55 participants were enrolled in the study. The distribution of participants according to the OCI categories was as follows: 21.8% reported no symptoms, 40.0% had low symptoms, 30.9% had moderate symptoms, and 7.3% had high symptoms.

### 3.1. Demographic and Clinical Characteristics

Among the 55 participants, most were older adults (*p*-value = 0.618). Additionally, 43.6% were males and 56.4% females. Females were more likely to report moderate-to-high OCI scores (51.6%) compared with males (20.9%), although this difference did not reach statistical significance (*p*-value = 0.128). Most participants were from Jeddah (58%). The distribution of OCI severity did not show statistically significant differences across residential areas (*p*-value = 0.567) or types of residence (apartments, villas, or others; *p*-value = 0.553) (Table 1A,B).

Tobacco use, including electronic cigarettes, was reported by 7.3% of the participants. However, no significant association was found between smoking status and OCI severity (*p*-value = 0.515) nor with the frequency of tobacco use (*p*-value > 0.99) (Table 1C).

Regarding clinical history, most participants (87.3%) had not undergone vision correction surgery, and 94.5% did not wear contact lenses. A history of chronic diseases other than those in this study’s exclusion criteria was present in 25.5% of participants, with no significant association between chronic disease and OCI severity (*p*-value = 0.083). A clinical diagnosis of DED was significantly associated with higher OCI severity (*p*-value = 0.024), with 66.7% of previously diagnosed patients reporting moderate to high symptoms (Table 1D).

### 3.2. Ocular Surface Parameters

The mean (SD) TBUT was 4.7 (3.2) s in right and 4.9 (3.1) s in left, both indicating tear film instability. The Schirmer test revealed a mean (SD) tear secretion of 17.4 (10.2) mm (right) and 18.4 (11.1) mm (left). No significant differences in TBUT or Schirmer values were observed when stratified by gender or age group (*p*-value > 0.05).

### 3.3. Bakhoor Use and Dry Eye Indicators

Most participants (90.9%) reported using incense. While 24% of those users had no symptoms, 36% had moderate-to-high OCI scores. No significant difference was found between incense use and OCI severity (*p*-value = 0.418). Among incense users, 50% reported using natural wood, which was significantly associated with moderate symptoms (*p*-value = 0.016). Powder-based incense use also showed a borderline association with increased symptom severity (*p*-value = 0.017) (Table 2A,B). Other forms of incense (natural gum, synthetic gum, sticks, paste) showed no significant link to the OCI category. Ventilation practices varied, with 42.3% not ensuring proper ventilation during incense burning, and 65.4% remaining in the same space while incense was burning. Neither ventilation practices (*p*-value = 0.163) nor remaining in the same space (*p*-value = 0.435) were significantly associated with OCI severity. Frequency of use, including daily or more than twice weekly exposure, was not statistically linked to OCI scores (*p*-value = 0.901) (Table 2A,B).

### 3.4. Subjective Ocular Symptoms

Comparative analysis using the Mann–Whitney U test showed a non-significant trend toward lower Schirmer scores among Bakhoor users (Left: *p*-value = 0.060; Right: *p*-value = 0.137). No significant differences in TBUT were observed between Bakhoor users and non-users, likely due to the small size of the non-user group (*n* = 5). Subjective dry eye symptoms were prevalent: 56.4% reported ocular dryness or discomfort within the past week, with 27.3% rating the severity as “high.” Ocular itching was reported by 61.8%, ocular fatigue by 52.7%, and ocular pain by 29.1%. Fluctuating vision was reported by 12.7% of participants. Among those previously diagnosed with DED (32.7%), median TBUT and Schirmer II test scores were not significantly different from those of undiagnosed participants (all *p*-values > 0.3). Similarly, the OCI categories (do not feel, low, moderate, high) did not demonstrate statistically significant differences in objective tear parameters (Kruskal–Wallis *p*-value > 0.05).

**Figure 1:**
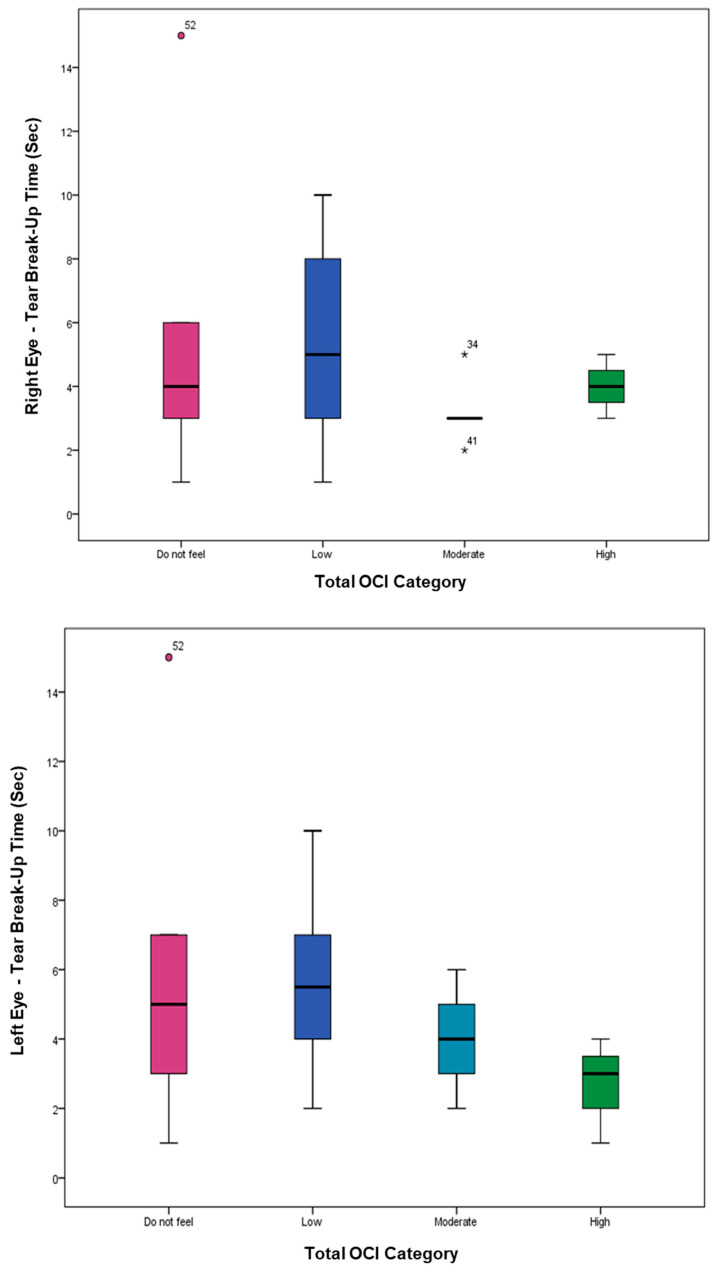
The whisker plot comparing OCI score group levels [do not feel (ruby color), low (navy color), moderate (turquoise color), and high (green color)] versus TBUT values per second (Sec.) for the right and left eyes using the median in the center line and the interquartile range (IQR) for box limits. The outliers are represented as asterisk (*).

The distribution of OCI categories showed that 21.8% had no symptoms, while 40.0%, 30.9%, and 7.3% reported low, moderate, and high symptoms, respectively. TBUT values displayed a downward trend with increasing OCI severity, with no statistically significant differences. Figure 1 illustrates the relationship between OCI score groups and TBUT (Right: *p*-value = 0.453; Left: *p*-value = 0.284).

**Figure 2:**
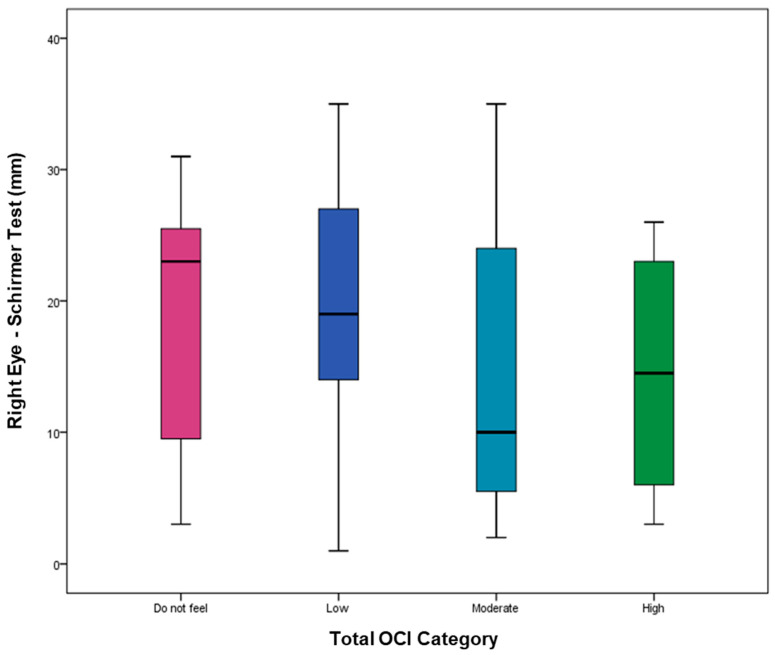

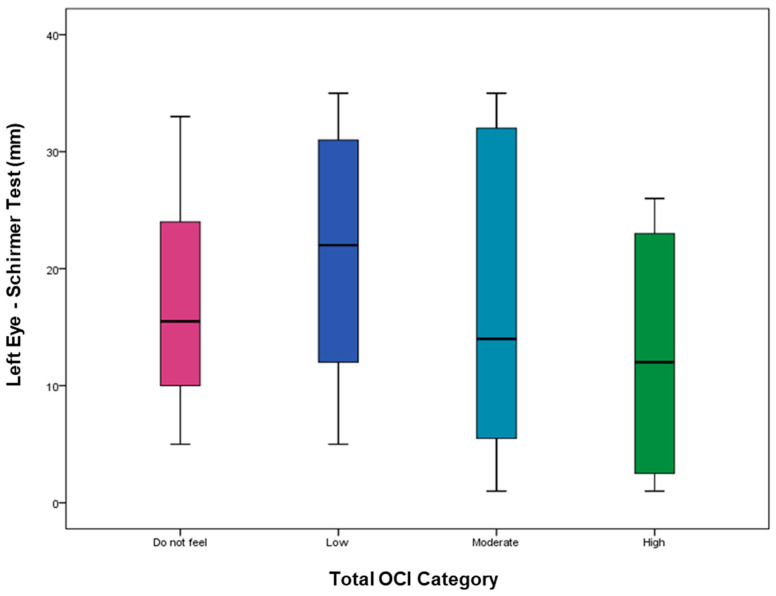
The whisker plot comparing OCI score group levels [do not feel (ruby color), low (navy color), moderate (turquoise color), and high (green color)] versus Schirmer score (mm) for the right and left eyes using the median in the center line and the interquartile range (IQR) for box limits.

The Schirmer values show a decreasing trend with increasing OCI severity, but there were no statistically significant differences. Figure 2 demonstrates the relationship between OCI score groups and Schirmer II test scores (Right: *p*-value = 0.403; Left: *p*-value = 0.372).

Among the 20 participants, all tear biomarker concentrations were inversely related to the OCI category, with no statistically significant differences in total protein, immunoglobulin E, and lactoferrin for both eyes (Table 3A–C). Table 3A shows that the mean (SD) of total protein (mg/mL) in the right and left eyes for high OCI was 7.19 (1.39) and 7.42 (0.91), respectively (*p*-values = 0.392 and 0.429). Table 3B shows that the mean (SD) of immunoglobulin E (ng/mL) in the right and left eyes for high OCI was 301.71 (55.97) and 301.71 (47.14), respectively (*p*-values = 0.381 and 0.137). Table 3C shows that the mean (SD) of lactoferrin (µg/mL) in the right and left eyes for high OCI was 163.77 (10.42) and 159.43 (1.68), respectively (*p*-values = 0.290 and 0.579).

## 4. Discussion

This study assesses DED as a common ocular surface disease of incense user patients using the OCI questionnaires. Here, the Schirmer score and TBUT values, which are DED diagnostic tools, were directly proportional to OCI scores.

The demographic finding that age and female gender were predominant factors among older adults is supported by prior reports from Saudi Arabia, demonstrating that assessing DED using the OCI score, as well as an international report by TFOS DEWS III [2,19,20,21]. This reinforces prior evidence supporting the OCI as a valid and reliable screening tool for ocular surface disease symptoms and underscores its potential utility in clinical settings [3].

Among environmental exposures, incense use emerged as a prominent risk factor. A substantial proportion of incense users reported moderate-to-high OCI symptoms, aligning with previous research linking incense smoke to ocular surface irritation and respiratory morbidity [6,22]. Natural wood incense, the most commonly reported type in our sample, demonstrated the strongest association with symptom severity, corroborating prior findings on its adverse effects on ocular health [15,23]. These results highlight the importance of considering cultural and lifestyle practices, such as incense burning, when evaluating environmental contributors to DED.

Ventilation practices also demonstrated a measurable influence on symptom severity. Participants reporting poor ventilation or remaining in enclosed spaces while burning incense exhibited higher OCI scores, reinforcing prior evidence that airflow and exposure levels are critical determinants of incense-related health risks [24,25,26].

To the best of our knowledge, this is the first study in Saudi Arabia to examine incense use in relation to ocular surface health, using subjective (OCI) and objective clinical assessments (TBUT and Schirmer II). Lower TBUT and Schirmer II values, which reflect impaired tear film stability and diminished tear production, respectively, were observed among incense users, further substantiating the hypothesis that incense smoke contributes to ocular surface dysfunction [27].

These findings have potential clinical implications. Tear film instability and reduced tear production, when left unaddressed, can progress to chronic ocular discomfort, visual disturbance, and long-term damage. Recognizing incense exposure as a modifiable environmental risk factor is therefore essential. Beyond its physiological impact, DED has broader consequences, impairing work productivity, limiting daily functioning, and adversely affecting psychological well-being.

Despite the non-significant results, these findings are essential to provide a baseline for future power calculations and to prevent publication bias regarding non-significant data. Moreover, this comprehensive assessment of ocular surface health concerning the diagnosis of DED was performed utilizing objective measures, specifically tear sample analysis, in comparison with the OCI category as an indicator of symptom severity. The findings indicate an indirect relationship between tear protein concentration and symptom severity. These results are consistent with prior research demonstrating that DED impacts tear protein concentrations [28], including total protein [29], IgE [30], and lactoferrin [31]. Such findings may inform more effective treatment strategies and patient counseling. This pilot study shows that while OCI remains a useful initial tool for subjective symptom assessment, combining it with objective clinical measurements and tear biomarkers offers a more detailed understanding of ocular surface health. The trends linking increased incense exposure, decreased tear film stability, and changes in proteins such as IgE and lactoferrin indicate a biological response to environmental irritants.

A limitation of this study was the number of non-users in the sample was small. This limited sample size reduces statistical power and increases the likelihood of type II error. The comparisons between incense users and non-incense users should be interpreted with caution and are considered to be exploratory rather than confirmatory. In addition, the small sample size is attributable to limited funding; however, this is an exploratory phase designed to inform future research. Due to the exploratory nature of this study, a convenience sampling technique was used, resulting primarily in adults and older adults, which may limit the generalizability of the findings to younger populations.

Future studies with stratified random sampling are required to validate these preliminary results. In addition, a recommended area for future research with larger and homogeneous groups is needed to validate these results, to determine the prevalence of dry eye among incense users, to explore several factors, including contact lens or eyeglasses users, environmental characterization, smoking, watering eye drops, ventilation area size, AC running, or duration of exposure within a cohort study design; and to investigate indoor air pollutants beyond incense. Finally, it is recommended that future research incorporate these markers in subsequent phases to compare the study biomarker values to the normal values in healthy people, and just report, for example, that lactoferrin was reduced in this population.

Despite the study’s limited sample size and heterogeneous cohort, which impose certain limitations, the implications derived from these preliminary findings indicate that clinicians should contemplate incorporating environmental history assessments into standard DED screenings. This involves utilizing subjective and/or objective tools to identify patients whose ocular symptoms are exacerbated by specific exposures, such as incense or particulate matter.

## 5. Conclusions

Differences exist between patients’ OCI levels and their ocular test outcomes, as well as between the concentrations of total protein, lactoferrin, and IgE in tear fluid, which may serve as predictors of the risk of developing DED symptoms or the disease itself. In addition, patients with high OCI levels show decreased values on ocular tests, including TBUT and Schirmer II, as well as decreased lactoferrin in tear fluid, while IgE in tear fluid is increased compared with patients with low OCI. Elevated levels are associated with exposure to household allergens originating from incense. The findings of this study suggest that OCI levels, along with ocular screening and tear fluid analysis, are indicative of the severity of DED.

## Figures and Tables

**Table 1:** (A,B) Patient data and demographic findings, (C) smoking status, and (D) dry eye disease findings.

	**(A)**		**Total OCI category**								
			**Do Not Feel**		**Low**		**Moderate**		**High**		***p*-Value**
		**Total**	**Mean**	**(SD)**	**Mean**	**(SD)**	**Mean**	**(SD)**	**Mean**	**(SD)**	
Age											
		44	48.9	(16.5)	44.8	(20.9)	52.9	(20.3)	40.7	(19.5)	0.618 **
	**(B)**		**Total OCI Category**								
		**Total**	**Do Not Feel**		**Low**		**Moderate**		**High**		***p*-Value**
			***n* = 12**	**%**	***n* = 22**	**%**	***n* = 17**	**%**	***n* = 4**	**%**	
Gender											
	Male	24	6	(25.0)	13	(54.2)	4	(16.7)	1	(4.2)	0.128 *
	Female	31	6	(19.4)	9	(29.0)	13	(41.9)	3	(9.7)	
Home address city/district											
	Jeddah	32	8	(25.0)	12	(37.5)	11	(34.4)	1	(3.1)	0.567 †
	Mecca	7	2	(28.6)	3	(42.9)	1	(14.3)	1	(14.3)	
	Bahra	5	2	(40.0)	2	(40.0)	1	(20.0)	0	(0.0)	
	Taif	6	0	(0.0)	3	(50.0)	2	(33.3)	1	(16.7)	
	AlBaha	1	0	(0.0)	0	(0.0)	1	(100.0)	0	(0.0)	
	Al Qunfudhah	1	0	(0.0)	1	(100.0)	0	(0.0)	0	(0.0)	
	Khamis Mushait	1	0	(0.0)	0	(0.0)	0	(0.0)	1	(100.0)	
	Tanomah	1	0	(0.0)	0	(0.0)	1	(100.0)	0	(0.0)	
	Al Majardah	1	0	(0.0)	1	(100.0)	0	(0.0)	0	(0.0)	
Type of home?											
	Apartment	31	9	(29.0)	10	(32.3)	10	(32.3)	2	(6.5)	0.553 †
	Villa	17	2	(11.8)	7	(41.2)	6	(35.3)	2	(11.8)	
	Other	7	1	(14.3)	5	(71.4)	1	(14.3)	0	(0.0)	
	**(C)**		**Total OCI Category**								
		**Total**	**Do Not Feel**		**Low**		**Moderate**		**High**		***p*-Value**
			***n* = 12**	**%**	***n* = 22**	**%**	***n* = 17**	**%**	***n* = 4**	**%**	
Do you smoke tobacco, electronic cigarettes, or others											
	No	51	11	(21.6)	19	(37.3)	17	(33.3)	4	(7.8)	0.515
	Yes	4	1	(25.0)	3	(75.0)	0	(0.0)	0	(0.0)	
If yes, how many times a week?											
	0	54	12	(22.2)	21	(38.9)	17	(31.5)	4	(7.4)	>0.99
	3	1	0	(0.0)	1	(100.0)	0	(0.0)	0	(0.0)	
	**(D)**		**Total OCI Category**								
		**Total**	**Do Not Feel**		**Low**		**Moderate**		**High**		***p*-Value**
			***n* = 12**	**%**	***n* = 22**	**%**	***n* = 17**	**%**	***n* = 4**	**%**	
Have you had vision correction surgery?											
	No	48	10	(20.8)	20	(41.7)	14	(29.2)	4	(8.3)	0.811 †
	Yes	7	2	(28.6)	2	(28.6)	3	(42.9)	0	(0.0)	
Do you wear contact lenses?											
	No	52	10	(19.2)	22	(42.3)	16	(30.8)	4	(7.7)	0.226 †
	Yes	3	2	(66.7)	0	(0.0)	1	(33.3)	0	(0.0)	
Do you have any chronic diseases that can lead to DED symptoms?											
	No	41	9	(22.0)	19	(46.3)	12	(29.3)	1	(2.4)	0.083 †
	Yes	14	3	(21.4)	3	(21.4)	5	(35.7)	3	(21.4)	
Have you been diagnosed with dry eye disease?											
	No	37	10	(27.0)	18	(48.6)	7	(18.9)	2	(5.4)	0.024 †
	Yes	18	2	(11.1)	4	(22.2)	10	(55.6)	2	(11.1)	

** ANOVA; * Chi-squared test; † Fisher’s exact test.

**Table 2:** Patient data (A) incense type and (B) incense utilization.

			**Total OCI Category**								
** (A)**		**Total**	**Do Not Feel**		**Low**		**Moderate**		**High**		***p*-Value**
			***n* = 12**	**%**	***n* = 22**	**%**	***n* = 17**	**%**	***n* = 4**	**%**	
**Do you use incense (Bakhoor)?**											
	No	5	0	(0.0)	2	(40.0)	2	(40.0)	1	(20.0)	0.418 †
	Yes	50	12	(24.0)	20	(40.0)	15	(30.0)	3	(6.0)	
Natural gum											
	No	37	6	(16.2)	15	(40.5)	14	(37.8)	2	(5.4)	0.251 †
	Yes	18	6	(33.3)	7	(38.9)	3	(16.7)	2	(11.1)	
Natural wood											
	No	30	7	(23.3)	16	(53.3)	4	(13.3)	3	(10.0)	0.016 *
	Yes	25	5	(20.0)	6	(24.0)	13	(52.0)	1	(4.0)	
Synthetic wood											
	No	48	8	(16.7)	20	(41.7)	16	(33.3)	4	(8.3)	0.190 †
	Yes	7	4	(57.1)	2	(28.6)	1	(14.3)	0	(0.0)	
Sticks											
	No	45	9	(20.0)	20	(44.4)	12	(26.7)	4	(8.9)	0.314 †
	Yes	10	3	(30.0)	2	(20.0)	5	(50.0)	0	(0.0)	
Synthetic gum											
	No	47	9	(19.1)	18	(38.3)	16	(34.0)	4	(8.5)	0.439 †
	Yes	8	3	(37.5)	4	(50.0)	1	(12.5)	0	(0.0)	
Paste											
	No	42	7	(16.7)	19	(45.2)	12	(28.6)	4	(9.5)	0.193 †
	Yes	13	5	(38.5)	3	(23.1)	5	(38.5)	0	(0.0)	
Powder											
	No	41	5	(12.2)	20	(48.8)	13	(31.7)	3	(7.3)	0.017 †
	Yes	14	7	(50.0)	2	(14.3)	4	(28.6)	1	(7.1)	
**(B)**			**Total OCI Category**								
		**Total**	**Do Not Feel**		**Low**		**Moderate**		**High**		***p*-Value**
			***n* = 12**	**%**	***n* = 20**	**%**	***n* = 17**	**%**	***n* = 4**	**%**	
Number of spaces utilized for incense											
	All home	46	11	(23.9)	15	(32.6)	17	(37.0)	3	(6.5)	0.129 †
	>3	6	1	(16.7)	5	(83.3)	0	(0.0)	0	(0.0)	
Do you ensure good ventilation when using Bakhoor, such as opening the windows, doors, and air conditioner (AC)?											
	No	22	5	(22.7)	12	(54.5)	4	(18.2)	1	(4.5)	0.163 *
	Yes	30	7	(23.3)	8	(26.7)	13	(43.3)	2	(6.7)	
Do you stay at the same place where you burned all Bakhoor?											
	No	18	4	(22.2)	8	(44.4)	4	(22.2)	2	(11.1)	0.435 †
	Yes	34	8	(23.5)	12	(35.3)	13	(38.2)	1	(2.9)	
Frequency of use per week											
	Never or rarely	7	1	(14.3)	4	(57.1)	2	(28.6)	0	(0.0)	0.901 †
	1–2 times a week	23	4	(17.4)	8	(34.8)	9	(39.1)	2	(8.7)	
	>2 times a week	21	6	(28.6)	8	(38.1)	6	(28.6)	1	(4.8)	

* Chi-squared test; † Fisher’s exact test.

**Table 3:** The assessment of tear protein concentrations for (A) total protein, (B) immunoglobulin E, and (C) lactoferrin.

		Total OCI Category								
		Do Not Feel (*n* = 6)		Low (*n* = 5)		Moderate (*n* = 7)		High (*n* = 2)		*p*-Value
		Mean	(SD)	Mean	(SD)	Mean	(SD)	Mean	(SD)	
(A) Tear total protein (mg/mL)										
	Right eye	5.84	(1.16)	6.42	(1.42)	5.38	(1.63)	7.19	(1.39)	0.392
	Left eye	5.91	(1.33)	6.50	(1.07)	6.11	(1.08)	7.42	(0.91)	0.429
(B) Tear immunoglobulin E (ng/mL)										
	Right eye	311.43	(72.18)	328.38	(24.36)	279.09	(31.03)	301.71	(55.97)	0.381
	Left eye	301.36	(54.90)	353.79	(47.41)	283.26	(44.02)	301.71	(47.14)	0.137
(C) Tear lactoferrin (µg/mL)										
	Right eye	157.98	(13.65)	157.03	(12.12)	147.04	(12.79)	163.77	(10.42)	0.290
	Left eye	157.37	(13.14)	156.93	(8.25)	150.35	(11.37)	159.43	(1.68)	0.579

## Data Availability

The original contributions presented in the study are included in the article materials. Further inquiries can be directed to the corresponding author for the restriction of privacy concerns.
